# Creating Safer Injecting Practices Through Community‐Led Harm Reduction: Lessons From People Who Use Steroids for the Alcohol and Other Drug Sector

**DOI:** 10.1111/dar.70044

**Published:** 2025-09-29

**Authors:** Timothy Piatkowski, Emma Kill, Geoff Davey, Monica J. Barratt, Jason Ferris, Suzanne Nielsen, Amy Peacock

**Affiliations:** ^1^ School of Applied Psychology and Griffith Centre for Mental Health Griffith University Brisbane Australia; ^2^ Queensland Injectors Voice for Advocacy and Action Sunshine Coast Queensland Australia; ^3^ Centre for Health Services Research, University of Queensland Brisbane Australia; ^4^ Queensland Injectors Health Network Brisbane Australia; ^5^ Criminology and Justice Studies, Social Equity Research Centre and Digital Ethnography Research Centre, RMIT University Melbourne Australia; ^6^ National Drug and Alcohol Research Centre, UNSW Sydney Sydney Australia; ^7^ Monash Addiction Research Centre, Eastern Health Clinical School, Monash University Melbourne Australia; ^8^ School of Psychological Sciences, University of Tasmania Hobart Australia

**Keywords:** abscess, anabolic–androgenic steroids, harm reduction, infection, injecting, safer injecting

## Abstract

**Introduction:**

Research on anabolic–androgenic steroid (AAS) use has addressed health outcomes, social dynamics, service access and risk management strategies. Yet little work has examined how these domains converge in injection practices, where community‐led knowledge and structural barriers shape harm reduction. This study aimed to explore people's experiences of injecting AAS, focusing on practices, challenges and sources of harm reduction knowledge, and examined how consumers develop safer injection methods through lived‐living expertise.

**Methods:**

Data were drawn from semi‐structured interviews with Australian AAS consumers (*N* = 25), including follow‐ups with 15 participants, resulting in 40 research interactions. These interactions examined safer injecting, harm reduction strategies and structural barriers. Analysis employed iterative categorisation, integrating lived‐living experience and a lens of prefigurative politics to develop knowledge into three theme‐categories.

**Results:**

Participants articulated that injecting, when done correctly, was perceived as a safer usage pathway. However, many participants lacked access to clear, evidence‐based injection guidance and acquired information through informal sources including online forums and peer networks. Some AAS consumers experienced bacterial infections and abscesses, highlighting the ongoing risks associated with injecting. Experienced consumers had developed practices, through experimentation, on site rotation, injection volume and hygiene to mitigate harms.

**Discussion and Conclusions:**

AAS consumers prefigure harm reduction through their community, yet informal learning is inconsistent. Peer‐led interventions which partner with trusted allies, including clinicians and health workers, can work toward reducing injecting‐related harms in the community; thus, moving the alcohol and other drug sector toward sustainable, collective care.

## 1 Introduction

The non‐medical use of image and performance enhancing drugs (IPED) such as anabolic–androgenic steroids (AAS) is a global phenomenon, with lifetime prevalence estimated at 3.3% [1]. The use of these substances is associated with significant physical (e.g., cardiomyopathy) as well as psychosocial (e.g., depression) issues [2]. While traditionally associated with bodybuilding, these substances are now used across diverse populations and motivations, including competitive athletes, fitness enthusiasts and gender‐affirming consumers [3, 4, 5]. AAS use is typically long‐term and highly structured, with a large proportion of consumers engaging in a combination of injecting and oral consumption of AAS (i.e., stacking), which reflect carefully planned usage strategies (e.g., cycles) [6, 7, 8]. As a result of these unique differences in consumption strategy, AAS remains poorly integrated into harm reduction responses for people who inject drugs, which were primarily designed around opioid and stimulant use [9, 10]. This study aimed to explore people's experiences of injecting AAS, focusing on practices, challenges and sources of harm reduction knowledge, and examined how consumers develop safer injection methods through lived‐living expertise.

Harm reduction responses to injecting drug use have historically centred on blood‐borne virus (BBV) prevention, needle and syringe distribution, and overdose intervention [11, 12, 13, 14]. These responses, designed primarily for opioid and stimulant use, are not always fit for purpose when applied to AAS consumers [10, 15, 16]. For example, while BBV risks remain relevant for people who inject AAS, prevalence appears substantially lower compared to people who inject other drugs. In an Australian cohort of people who use AAS (*N* = 60), Larance et al. [17] found lower HCV prevalence rates (5%) among people injecting AAS while more than half reported redness, soreness, or swelling at injection sites, with one in five of those having developed an abscess or infection. In another Australian study of 156 people who inject AAS, HIV and HCV antibody prevalence was 1% and 9%, respectively [18], whereas HIV and HCV prevalence among people who inject drugs is estimated at around 15% and 38%, respectively [19]. However, testing was relatively infrequent in the AAS group, with only 33% tested for HIV and 34% for HCV in the past year [18]. Other injection‐related harms were more commonly reported, with 37% experiencing injection site redness and 4% reporting abscesses [18], potentially exceeding the global estimates of recent skin or soft tissue infection (31%) among people who inject drugs [19]. Lastly, among Australian AAS consumers, 6%–29% report sharing AAS multi‐use vials and containers [17, 18]. These contrasting findings highlight that BBV prevalence does exist; however, people who inject AAS also face other significant risks related to injecting injuries, unsafe practices and under‐utilisation of health services.

A growing corpus of literature highlights how AAS consumers actively seek out and share information [20, 21], develop community‐led strategies [22, 23] and rely on community networks for guidance on health, dosage and harm reduction [24, 25]. Studies have documented the central role of online forums, gym communities and trusted peers in shaping use practices [26, 27]. Research also shows that AAS consumers often express mistrust toward mainstream health services due to previous experiences of stigma, dismissal or poor clinical knowledge [28, 29], and this is evident in Australia specifically [3, 15, 30]. These structural barriers are compounded by a lack of workforce training on how to support people who use AAS in non‐medical contexts [30], and particularly among the health workforce involved in needle and syringe programs (NSP) [15, 31, 32]. In Australia, conventional public health models typically include NSPs, BBV testing and information dissemination primarily oriented toward people who inject psychoactive drugs [4, 15, 33]. These services typically focus on BBV and overdose prevention. Some programs, such as those operated by Queensland Injectors Health Network and select primary health networks, are beginning to offer AAS‐specific resources [15, 34]. However, these efforts remain unevenly distributed and inconsistently integrated into mainstream alcohol and other drug or sexual health services, and often do not account for the unique needs, motivations and usage patterns of people who use AAS [35].

While a small number of harm reduction interventions, such as the AAS testing programs in Australia [36] and Switzerland [37], have started to engage this population in meaningful, peer‐informed ways [38], these remain exceptions rather than the rule. There is limited empirical research exploring how people who inject AAS navigate harm reduction in practice [9, 33, 39], particularly in the absence of institutional support. One aspect of harm reduction that is often overlooked in this context is the role of injection itself. Intramuscular injections of AAS bypass first‐pass metabolism and thereby may reduce the direct hepatic and renal burden [40]. This may explain why many consumers report using injectable AAS more often than oral compounds [36, 41], as consumer‐led messaging has acknowledged the risks of hepatic strain from oral use [42]. Given that oral AAS can cause rapid enzymatic changes within days or weeks [43], it follows that consumers may perceive injections as a safer and more reliable option for longer term usage. This contrasts with harm reduction orthodoxy in other drug‐consuming populations, where injecting is positioned as the highest‐risk route of administration [11, 44, 45, 46]. Yet, current messaging rarely acknowledges these differences [47]. In the absence of sustained institutional attention, people who use AAS have built their own harm reduction strategies, drawing on lived‐living experience and community care. Therefore, this study aimed to explore people's experiences of injecting AAS, focusing on practices, challenges and sources of harm reduction knowledge, and examined how consumers develop safer injection methods through lived‐living expertise.

### 1.1 Approach

Harm reduction is not just a set of interventions, it is a political and ethical framework grounded in principles of pragmatism, dignity and community‐leadership [48, 49, 50, 51, 52]. We adopt a prefigurative politics [53] lens to examine how harm reduction for people who inject AAS can be reimagined beyond conventional public health models. Carl Boggs [53] defined prefigurative politics as the embodiment of social relations, decision‐making, culture and human experience that movements seek to bring about. Unlike protest movements that confront dominant regimes, it enacts a vision of a better world through practice, creating the care, safety and infrastructures we seek now, rather than waiting for institutional validation. Rooted in late 19th‐ and early 20th‐century anarchism, figures like Kropotkin [54] argued that movements should reflect their goals in their practices. However, the recent effervescence of prefigurative politics reflects a widespread disenchantment with institutional models and the emergence of alternative forms of social organisation [55, 56]. For people who use AAS, this disenchantment with traditional medical and legal systems [4] is mirrored in the refusal to accept the exclusion of their needs from mainstream healthcare systems [57]. Instead, many community‐led networks have developed their own societies in which harm reduction is enacted specific to communities using AAS [22].

These practices are not always explicitly prefigurative but contribute to broader transformation. In AAS use, safer practices emerge from necessity and community responses to institutional neglect [3, 24], driven by a desire for change. While the population of people who inject AAS is not homogenous, and some groups, particularly English‐speaking men accessing urban NSPs, report relatively good engagement with services (e.g., nearly two‐thirds in one Sydney study had disclosed use to a doctor) [35], others face considerable barriers. These include consumers from culturally and linguistically diverse backgrounds or those newer to injecting, who often rely on informal sources of information and are less likely to trust or access medical advice [15, 30]. Therefore, prefigurative politics in this context speaks to how marginalised subgroups within the broader AAS‐using population negotiate healthcare exclusion, stigma and power imbalances. It involves a rejection of traditional authority structures and an assertion of autonomy over one's health and wellbeing. The political nature of these practices lies in their direct engagement with, and reworking of, the structures that marginalise and stigmatise consumers [23, 29, 58], embodying a vision for a more equitable and responsive system in the present.

By framing harm reduction as an ongoing process of *world‐building*, this study contributes to a growing body of scholarship that recognises drug use not as an inherently deviant or pathological act but as something embedded in complex social, material and political networks. For AAS consumers, safer use practices, including safer injection, are actively being created and sustained through community‐led infrastructures of care. These prefigurative practices resist punitive policies and offer a radical, community‐led alternative for safer, more just futures.

## 2 Methods

### 2.1 Design and Ethics

This research is situated within a participatory exploratory approach, engaging with communities who use AAS in ways that reflect the co‐creative principles of prefigurative politics. The study draws on initial and follow‐up interviews with participants and recognises them as active collaborators in the production of knowledge and meaning‐making. Ethical approval was granted by the Griffith University Human Research Ethics Committee (Approval: 2023/784).

### 2.2 Sampling and Recruitment

The lead author is a peer‐researcher with lived‐living expertise in IPEDs. Participants were recruited through the lead author's extensive, longstanding networks within the Australian AAS community, which include connections with commercial gymnasia, powerlifting and strongman gyms and supplement stores, as well as individual AAS consumers and coaches across multiple states and territories. Recruitment also involved collaboration with community organisations, such as the Queensland Injectors Health Network and Queensland Injectors Voice for Advocacy and Action who are active in advocating for harm reduction for people who use AAS. These networks facilitated access to a diverse group of AAS consumers, including individuals with varying levels of engagement in peer support and harm reduction activities. Participants were required to be residing within Australia, at or over the age of 18 years, and currently or recently (i.e., within 12 months) used AAS. The lead author shared study details through word‐of‐mouth in a purposive approach. Recruitment was voluntary and included those who expressed interest through direct contact, social media promotion and word of mouth. Once participants had been identified and interviews had been conducted, the lead author requested that the participants share the study details with their peers, known as snowballing. Informed consent was obtained verbally before each interview and was achieved through the interviewer providing a thorough overview of the study, its aims and objectives.

### 2.3 Data Collection

The initial interview was designed in collaboration with the AAS community, via the Queensland Injectors Voice for Advocacy and Actions' ‘Steroid Advisory Group’. The lead author met with members of the group informally to ensure the interview guide was culturally sound. Example questions from the first interview included: *How serious do you consider the potential health risks associated with using AAS?* and *Do you believe you are at risk of experiencing negative health effects from using anabolic–androgenic steroids (AAS)?*. Prompt included: *Can you provide specific concerns?* The lead author, recognised in the community as a person with lived‐living experience and who advocates for health equity for AAS consumers, made initial contact with each participant, introduced himself (if not previously known) and explained the purpose of the research. This initial peer‐to‐peer discussion facilitated a culturally safe approach and was performed in‐person, online via direct message, or via phone. The lead author then matriculated participants through to a research assistant via online introduction. The assistant then booked an agreed time to conduct audio‐recorded online interviews via Microsoft Teams. Initially, 25 interviews were conducted in this manner.

As these initial interviews progressed, narratives of injection‐related injuries, bacterial infections and difficulties accessing medical care emerged as key components of participant experiences and were accordingly documented. Documenting the concerns of the community as they perceive them was a fundamental aspect of the community‐led research design we followed. Accordingly, we adapted the interview guide in response to participant insights, ensuring that the research remained grounded in the priorities of those with lived‐living experience. The identification of injection‐related harms as a key concern demonstrated the salience of these issues within the community. It also underscored the necessity of harm reduction initiatives that are responsive to the distinct risks and knowledge needs of people who inject AAS.

To investigate these issues further, follow‐up interviews were conducted with 15 participants. Participants were asked directly about their experiences: *What have been your experiences with injecting?*. They were also asked about complications: *Have you experienced any infections or injuries from injecting?* and *How were your experiences accessing medical treatment for these?*. To explore broader structural factors shaping harm reduction, participants were prompted: *How does social stigma around steroid use affect your ability to practice safer use?* and *Do you think there is enough safe injecting information available?*. Finally, to examine the specificity of harm reduction needs, participants were asked: *As compared to other people who inject drugs, what type of information do people who use steroids need?*. These questions aimed to capture the lived‐living experiences of injecting, the accessibility of medical support and the structural and informational barriers affecting safer AAS injecting. Collectively, between June 2024 and January 2025, 25 people who inject AAS participated in 40 online semi‐structured interviews (mean length = 55 min, range = 32–87 min).

### 2.4 Data Analysis

A research assistant transcribed the interviews verbatim and then de‐identified them. Data collected in both phases were utilised for analysis. The lead author conducted a systematic, inductive line‐by‐line analysis using iterative categorisation to refine and code the data [59]. This process identified and developed codes related to safer use and injecting practices. Inductive thematic sufficiency was achieved [60], and recurring themes were organised into higher‐order concepts, forming a structured framework for presenting preliminary findings.

As part of the identification and development of thematic categories, the lead author recognised experiential patterns voiced by participants that resonated with his own lived‐living experience of AAS injecting. These included relationality with infections, seeking or avoiding medical care and navigating the lack of stigma‐free and contextually relevant resources. Drawing on this peer‐informed lens enabled a deeper attunement to the nuances of participant narratives. This positionality aligns with frameworks of ‘evidence‐making’ and ‘thinking with’, which value lived‐living experience not only as data but also as a method of knowledge exchange [38]. In this way, the analysis was also shaped by the emotional and biographical intersections between participants' stories and the researcher's own experience [10].

To ensure that this positionality enriched rather than overpowered interpretation, the lead author engaged in critical reflexivity and collaborative discussion throughout the analytic process. Once potential theme‐categories were developed, the research team, including the perspectives of peer researchers (T.P., E.K., M.J.B.), those with lived‐living experience of AAS injecting (T.P.) and trusted academic and clinician‐practitioner allies (A.P., J.F., S.N., G.D.), collectively examined the findings. This approach challenged assumptions, surfaced tensions and ensured that the analysis reflected diverse forms of expertise. The key findings are presented below within three thematic categories: negotiating injecting risk; injection literacy and the gaps in practical education; and community‐led prefiguration of safer use.

## 3 Results

Semi‐structured interviews were conducted with 25 participants (*Mean* age = 35.8 years, range = 22–49 years; 20 men, 5 women). Of these, 15 participants (*Mean* age = 36.3, range 28–45 years; 11 men; 4 women) participated in a follow‐up interview. All participants reported using a variety of injectable (e.g., testosterone, trenbolone, drostanolone) and oral (e.g., oxandrolone, fluoxymesterone) compounds at the time of the interview.

### 3.1 Negotiating Injecting Risk

Participants described a range of complications, from injection site infections to more severe reactions that could result in long‐term health consequences. Several participants reflected on their experience with abscess formation, describing how improper injection techniques or site selection could lead to painful and serious infections:

Felix (32, man): 'Sometimes you get a sore injection site. There was 1 pretty bad abscess in my thigh, my outer quad. It was like a pool under the skin in between the muscle and the skin. It was just all full of puss.'

For some, the risks of incorrect administration extended beyond infections, particularly when substances were mistakenly injected into a vein. The thick, oil‐based composition of many AAS, such as testosterone, meant that intravenous injection could trigger severe physiological responses:

Rose (45, woman): 'I know a few people who the very first time they tried, they put it straight into a vein. So like the viscosity of testosterone, it's literally an oil compared to blood. So those dudes sort of spent like 40 min fighting for their life, coughing once it hits the lungs.'

Felix (32, man): 'You know when you've nicked a vein, ‘cause you can taste it in your mouth. You'll get a chemically or an oily taste come to your tongue.'

The oil‐based formulations pose unique risks not typically encountered in other injecting drug use, complicating harm reduction messages that often focus on BBV transmission. This also illustrates how unfamiliarity with these substances can amplify harm beyond what is commonly understood within standard injecting drug use education. Beyond infections and vascular complications, nerve damage was another under‐recognised risk, often linked to limited knowledge about safe injection practices. One participant recounted a case where injecting into an inappropriate site resulted in lasting neuropathy:

Grace (28, woman): 'I know a person who used an odd section of their leg for quite a large volume [of AAS]. Now they have like a neuropathy on the side of their leg. They've got like no feeling whatsoever there and it never came back. Nerve damage is probably one of the bigger things that I think occurs to people ‘cause they don't necessarily know where they [nerves] are.'

This example draws attention to the structural knowledge gaps and lack of accessible, targeted education around injection site anatomy for AAS consumers. For those using specific AAS such as trenbolone, additional risks emerged. ‘Tren cough’, a phenomenon where the compound provokes an immediate and severe respiratory reaction, was described as both distressing and dangerous:

Leo (32, man): 'The first time someone gets tren cough, they think they might, die like it's horrible because as soon as you draw that needle out, it's basically instantaneous. Sometimes you don't even get a chance to get the needle out. I have heard of people that have snapped off small needles because of coughing.'

The physiological reaction known as ‘tren cough’ illustrates the intersection between pharmacological properties and user experience, highlighting risks that are compound‐specific and not easily generalised across AAS use. These accounts illustrate the real and often poorly understood risks associated with injecting AAS. Many participants viewed bacterial infections as a greater risk than the pharmacological profile of the substances. Many participants stressed that these infections were their primary concern.

Samson (39, man): 'I would be much more concerned about bacteria being in it [steroid vial] than if it's overdosed, underdosed or even slightly wrong drug. Because that's where the real risk is in terms of actual health. You can lose your freaking leg. You can lose a chunk of your quad [quadriceps] or your glute [gluteus maximus]. It's way more scary to me than if I used weak testosterone or strong testosterone.'

This prioritisation of bacterial infection risk over pharmacological risk suggests that users are highly attuned to immediate, tangible harms that have direct physical consequences. Building on this, many participants highlighted that when infections become severe, they often prefer seeking advice from their community rather than formal healthcare providers:

Leo (32, man): 'I would say a lot of side effects people get are self‐induced when it comes to cleanliness and hygiene. But, it's just part of the process now. You eat, you train, you pin [inject]. Nothing exciting about it anymore. It's only when it gets to the point of a severe infection that they're willing to go to the emergency room or a doctor. They'd rather get their advice from the forum, take a photo and put it on the forum.'

This account demonstrates the extent to which injecting practices are integrated into participants' embodied routines, treated as banal rather than exceptional. At the same time, the preference for seeking advice within peer networks, even in response to complications such as infections, reveals a dual dynamic: the practical utility of community knowledge and the enduring disaffection with formal health systems. Further illustrating this disconnect, several participants noted the minimal effort many AAS consumers invest in seeking professional treatment:

Oliver (37, man): 'Most of them won't go and get medical treatment, or if they do, they go to the doctor and just ask for an antibiotic. That's it. They don't go and get it drained or they try to drain it themselves.'

Such practices, while born out of necessity, also demonstrate the community's capacity to self‐organise and model safer practices in the absence of culturally safe institutional frameworks. The reluctance to pursue more rigorous treatment, even in the face of potentially dangerous infections, underscores a systemic issue where the healthcare environment is perceived as unsupportive or judgmental. For instance, several participants recounted the difficulty of accessing adequate care for an infection.

Asher (27, man): 'The hard part was going to a doctor and trying to get antibiotics. Because their biggest thing was I shouldn't be using that. And like, yeah I get that, but I have an infection now. Please help me.'

This exemplifies the tension between healthcare providers' moralising attitudes and consumers' immediate health needs, contributing to delayed care and worsening outcomes. Further, the risk associated with intramuscular injections was contrasted with the representation of information at NSPs, which primarily addressed viral transmission rather than bacterial infection:

Oliver (37, man): 'There's plenty on intravenous [injecting] at like needle exchanges. It'd be great if they had stuff specifically for intramuscular because the risks are more to do with bacterial infection than viral infection. So, the chances of hepatitis and AIDS are minimal because no one is sharing needles.'

While harms like abscesses or bacterial infections are not unique to AAS use, participants associated them specifically with the practice of intramuscular injection, often conducted in informal or unsupervised settings without access to tailored guidance or clinical support. However, this issue goes beyond missing information. Rather, it reflects a broader absence of structurally and socially attuned harm reduction that acknowledges the distinct injecting practices, service barriers and stigma that shape how people who use AAS navigate care. As our findings suggest, safer practices are not only about technical knowledge, but about fostering trust, relationality and meaningful inclusion within health systems often designed without this population in mind.

### 3.2 Injection Literacy and the Gaps in Practical Education

Several participants compared the risks associated with different methods of administration.

Douglas (28, man): 'The injectables are the lesser of two evils when it comes to liver and kidney health. But people will take Anavar [oxandrolone] because it's easy, they can just pop a pill and take Anadrol [oxymetholone] and Superdrol [methasterone]. But they are some of the harshest on the liver.'

However, this comparative safety is contingent upon proper injection practices as well as access to community‐informed support, reinforcing the importance of relational and contextually appropriate education. Many participants emphasised the importance of literacy regarding all types of IPED injection techniques, underscoring the need for ongoing availability of education and support.

Rex (41, man): 'It doesn't hurt to refresh people's memories about how to do their injections and which certain sections to do their injections. I was talking to a bloke in the gym the other day and he was like, oh, my leg's so sore. I was like, why? And he's like, I put my growth hormone into my muscle tissue. And I was like, what the fuck would you do that for? Like, growth hormone is a subcutaneous injection.'

Many participants also highlighted that, while digital resources are widely available, they often lack the clarity and situated relevance needed for IPED‐using communities beyond AAS, leading to misinformed practices and avoidable health risks.

Damien (37, man): 'The Internet has lots of information, but I guess a lot of it's just random people who put information up.'

Damien's critique exposes the uneven quality of publicly available resources, suggesting that the democratic availability of information online does not equate to reliable or safe guidance. It reflects a broader challenge in health education where misinformation proliferates, particularly in niche or stigmatised communities. His sentiment was echoed by other participants, who lamented the inadequacy of many instructional materials:

Zane (31, man): 'I've found PDFs. But not actually going through and showing on a person the injection site and how it's done. Or it's done on like teddy bears or oranges […] you go to YouTube [and] it's not actually showing a person going through the process.'

Here, Zane points to a significant disconnect between abstract or generic educational formats and the practical, situated learning needs of consumers. The use of inanimate models or partial demonstrations fails to translate fully into user confidence or competence, indicating the necessity of more immersive, peer‐informed training approaches. These reflections revealed a gap between theoretical knowledge and practical, demonstrative guidance. The reliance on generic digital content leaves many AAS consumers without the hands‐on instruction necessary for safer injecting. Many participants underscored the foundational issue of injection anxiety and the necessity for practical education.

Alexander (31, man): 'The biggest barrier's like people don't want to inject or they don't know how to inject. They're scared of injecting. I think just education on how to actually do it all. I think people are afraid of even just YouTube. Like if there was something that would be accessible without the whole kind of stigma.'

Here, the psychological dimension of injection practices is foregrounded: fear and anxiety are significant barriers that education alone must address. The mention of stigma reflects how societal judgements compound these fears, creating barriers not just to information access but to practical skill acquisition. Participants underscored that the lack of accessible, stigma‐free education on injection techniques contributes to significant risks and uncertainties. This highlights the need for educational interventions that are both technically informative but also embedded in trust‐based relationships that foster confidence and inclusion.

### 3.3 Community‐Led Prefiguration of Safer Use

Trust and community identity strongly influence the dissemination of safer injecting practices. Many participants remarked on the relative credibility of peer instruction:

Alexander (31, man): 'So if some big roided up dude was showing me how to inject, I'd trust him more than just like some fucking nerd doctor.'

Alexander's statement underscores the profound role of peer credibility, where experiential knowledge and embodied familiarity often trump formal medical authority. The credibility of peer instruction reinforces trust within the community and ensures harm reduction practices are culturally resonant and practical. However, reliance on peers presents challenges when access to accurate information is inconsistent, raising questions about how credibility is established and the risk of misinformation. Here, a tension emerges between the benefits of community‐led education and the potential for uneven or inaccurate knowledge transmission. Beyond fostering trust, community‐led education plays a crucial role in transmitting technical knowledge, including strategies to mitigate injection‐related harms. One such example is the importance of site rotation, which several participants identified as essential for preventing long‐term tissue damage:

Penny (33, woman): 'A lot of people get happy with the one area. What they don't realise is when you continuously inject in one place, you start to build scar tissue.'

Penny's reflection highlights a nuanced understanding of cumulative physical harm, showing that knowledge shared through peer networks often includes practical, long‐term health considerations rather than just immediate effects. Her observation emphasises the importance of proper site rotation to prevent long‐term tissue damage. It also points to the need for comprehensive guidance that addresses both the immediate and cumulative risks of injection practices. Practical advice on site rotation further illustrates the importance of experiential knowledge. Several participants provided detailed guidance on the necessity of rotating injection sites to avoid scar tissue:

Ben (40, man): 'For example, someone who pins [injects] daily, every single day. If you just go 1234 like this [points alternating each arm], you'll have so much scar tissue and such sore shoulders. You have to start using OK, shoulder, shoulder, glute, glute, quad, quad. You have to use all of the sites.'

This reflects an adaptive knowledge system that accounts for frequency and bodily impact, suggesting that peer networks act as dynamic, context‐sensitive knowledge hubs. However, another layer of complexity arises from individual differences in body composition and how these affect injection volumes and techniques. Several participants explained the distinctions between various injection methods and the importance of using appropriate equipment:

Asher (27, man): 'I'm sitting at 150 kilos [kilograms] and you know the size of that glutes [gluteus maximus] are very different. I know I could put more than 3 ml [in] [be]cause I've done 5 ml. What happens to the girl who's 50 kilos, who's never done it [injected] before going. “Oh, I should be able to put 3 ml in here”.'

Many participants highlighted the variability in safe injection practices based on individual physiology, suggesting that a one‐size‐fits‐all approach to education is insufficient. Dante highlighted how peer knowledge is highly individualised, reflecting embodied differences that challenge standardised approaches:

Dante (39, man): 'There are differences between the gauges and needles kind of thing what you would use [and] where you would use it. So, like I wouldn't use the insulin needle on my glute for example, just because of how much skin and fat is in between there. But I could do it on my delt [deltoid; shoulder].'

In addition to technical knowledge, community‐led harm reduction was shaped by deeper interpersonal connections and shared cultural values. These relationships fostered trust, belonging and a sense of collective responsibility. For many, the act of injecting was not an isolated or purely medicalised practice, but embedded within broader social rituals and affirming interactions of IPED‐using communities. As one participant explained:

Douglas (28, man): 'You end up in these kind of crews, and it's not just about gear or pinning, it's like […] looking after each other. If someone's new, you show them how to do gym, how to eat and meal prep, and if they starting jabbing then you show them that too […] It's just how we do things.'

Such accounts point to how harm reduction was experienced not just as the transmission of safer use techniques, but as an embodied, social practice grounded in mutual care. These micro‐communities acted as support systems, where people felt seen, affirmed and accountable to one another. In this way, harm reduction exceeded the boundaries of risk mitigation, functioning as a relational, emotionally meaningful practice that builds alternative cultures of care. These insights expand our understanding of prefigurative politics, highlighting how everyday practices of injecting and instruction reflect a broader effort to craft supportive, inclusive spaces that challenge the alienation often found in formal healthcare systems.

## 4 Discussion

This study set out to explore people's experiences of injecting AAS, focusing on practices, challenges and sources of harm reduction knowledge, and examined how consumers develop safer injection methods through lived‐living expertise. Through the lens of prefigurative politics [53], we sought to reimagine harm reduction not as a top‐down strategy, but as a process already being enacted within communities of people who use AAS. Our findings indicate that severe bacterial infections and other injection‐related complications are an important concern among people who inject AAS. Participants frequently described experiencing injection site injuries, such as abscesses, localised inflammation and signs of infection, with many reporting that these issues were widespread across their community. These observations resonate with previous research [23, 58], suggesting that enhancing injection literacy and harm reduction strategies is urgently needed. Participants consistently reported an absence of accessible, culturally appropriate and stigma‐free guidance on injection technique. Rather than being pushed to seek alternatives, many actively chose to rely on peer‐sourced knowledge and self‐treatment strategies, viewing these as more trustworthy, relevant and reflective of their needs. In this way, participants are not merely filling gaps left by formal systems; they are actively shaping harm reduction environments through collective learning and innovation, chiming with previous work [55]. This is precisely where prefigurative politics comes into play: communities are not waiting for institutions to validate their needs; they are building the kinds of health‐promoting futures they want to see, right now.

Harm reduction, then, should not be seen solely as a mitigation strategy. As we argue throughout this paper, the dominant paradigm frequently positions safer use, particularly injecting, as a morally compromised but necessary concession, reinforcing stigma and pathologising AAS consumption [29, 61]. Our findings directly challenge this framing. Participants in our study actively engaged in detailed, thoughtful injection practices not because they were constrained by fear or risk, but because they were invested in care, community knowledge and bodily autonomy. In this way, community‐led harm reduction practices represent intentional efforts to build new, shared norms around safety, knowledge exchange and mutual accountability. These are the hallmarks of prefigurative politics in action [56], where communities model the kind of care and systems they want to see, even in the absence of institutional support. Yet, despite this transformative potential, many of these initiatives continue to operate in silos, unsupported by broader healthcare structures. Without integration into formal systems, the insights and innovations of community‐led harm reduction remain under‐recognised and under‐utilised.

People who use drugs often establish spaces of collective empowerment and self‐regulation [62]. Previous research has found that AAS community‐led initiatives derive their strength from peer‐sourced information, even though significant gaps persist in accurate and accessible harm reduction knowledge—both within peer networks and the broader medical and academic literature [22]. By drawing on prefigurative politics, our study demonstrates that community‐led harm reduction is not merely a stopgap response to institutional neglect. Rather, it represents a transformative effort by AAS consumers, one that can be leveraged by researchers and healthcare professionals to create more culturally relevant strategies. For instance, in our study, detailed peer advice on technical matters, such as the importance of proper site rotation to prevent scar tissue and maximise AAS absorption, illustrates how experiential knowledge is transferred within the community, filling a void left by formal healthcare systems. Our approach demonstrates that by integrating targeted, community‐led educational interventions with broader healthcare support, we can transform injection practices from damage control to proactive, community care.

As scholars have indicated, Australia's current harm reduction responses have struggled to address the complexities of IPED consumption for over a decade [9]. We build on this evidence and indicate that there are still critical gaps in injection literacy for intramuscular and subcutaneous injecting. For instance, many participants expressed frustrations with generic online materials that fail to provide detailed, practical guidance on safer injection techniques, an issue compounded by the persistent stigma surrounding injection practices. While many participants described injecting as a routine or mundane part of their enhancement practices, dominant health narratives often position injecting as extreme, deviant or inherently risky. So then, by deradicalising the act of injecting itself, not for those who inject, but for the institutions and publics that continue to treat it as transgressive, we can begin to address the actual needs of communities who use AAS. This reframing emerges through the mundane, skilled and socially embedded accounts provided by participants. While approaches rooted in lived‐living experience have been transformative in filling gaps in injection literacy and offering practical advice, they are not without limitations. As we have seen, the reliance on informal, often fragmented information can also lead to risks, such as injection‐related injuries and other complications. Given the complexity of the health, physiological and pharmacological aspects managed in AAS use, there is a clear opportunity for these initiatives to be co‐developed in partnership with trusted allies such as clinicians, health workers, policymakers and other relevant stakeholders. This collaborative approach would ensure programs are informed by lived‐living experience while also benefiting from additional expertise and systemic support for a more comprehensive and effective response.

The practical implications of our study are significant. Integrating AAS health services such as NSPs, pharmacies and healthcare providers is important for enhancing the provision of accurate, evidence‐based harm reduction information in these settings. Our findings point to a clear opportunity to coordinate and develop peer‐led educational materials that truly meet the needs of communities using AAS. Such materials should not only provide technical guidance on safer injecting practices but also be led from a position of lived‐living expertise alongside the health workforce and clinicians who have demonstrated allyship with this community. In partnership with community‐led organisations, these educational resources could include live demonstrations, interactive workshops, and multimedia content that truly reflect the lived‐living experience of AAS use and accommodate diverse body types, experiences and contexts within communities. By viewing harm reduction as an active, community‐driven process of world‐building, our study highlights the transformative potential of peer‐led initiatives. These initiatives mitigate immediate risks and also lay the groundwork for a more culturally resonant, sustainable and empowered peer‐led model of care. Importantly, this goes beyond merely disseminating safer injecting tips and instead affirms the legitimacy of AAS use and the expertise embedded within these communities.

By foregrounding prefigurative politics, our study moves the discussion beyond institutional gaps and toward community‐based solutions. Harm reduction, in this frame, is not just about preventing harm; it is about enabling agency, fostering solidarity and building new cultural logics of care. Community‐led initiatives for AAS, as described in this study, exemplify this future‐facing orientation: they mitigate immediate risks while offering alternative, affirming ways of engaging with health. These are not just responses to exclusion; they are practices of resistance, care and transformation.

### 4.1 Limitations

While our study provides valuable insights into the risks of AAS injecting and the transformative potential of community‐led harm reduction, its findings may not fully capture the diversity of AAS consumers. Our sample was drawn from Australia, via the lead author's networks, potentially limiting perspectives from those less engaged in peer networks. Additionally, this study did not explicitly explore participants' motivations for AAS use and, further, did not explore intersections between gender identity and AAS consumption choices. These factors may influence experiences and needs, particularly within the framework of prefigurative politics and culturally appropriate interventions. Future research should explore broader demographic and geographic contexts to ensure harm reduction interventions are inclusive and responsive to the full spectrum of IPED use beyond AAS.

## 5 Conclusion

While injecting AAS carries risks, these can be significantly reduced with the right knowledge, resources and support. This study does not suggest that injecting is inherently safe, but rather that there is a critical opportunity to improve safety through culturally resonant initiatives. By reimagining harm reduction to centre lived‐living expertise, providing accessible, stigma‐free guidance and fostering partnerships between peers and allied clinicians and health workers, we can work toward a proactive, community‐led model of care for this underserved community. Doing so requires investment in initiatives that offer engaging and culturally relevant education.

## Author Contributions

**Timothy Piatkowski (TP):** conceptualisation, funding acquisition, data collection, data analysis, manuscript writing and review. **Emma Kill (EK):** funding acquisiton, data analysis, manuscript review. **Geoff Davey (GD):** data analysis, manuscript review. **Monica J. Barratt (MJB):** data analysis, manuscript review. **Jason Ferris (JF):** data analysis, manuscript review. **Suzanne Nielsen (SN):** data analysis, manuscript review. **Amy Peacock (AP):** data analysis, manuscript review.

## Conflicts of Interest

G.D. is the CEO of Queensland Injectors Health Network. E.K. is the CEO of Queensland Injectors Voice for Advocacy and Action, and T.P. is on the Board of Directors of the organisation. A.P. is supported by a National Health and Medical Research Centre Investigator Grant (1174630). S.N. is supported by a National Health and Medical Research Council Investigator Grant (2025894).

## Data Availability

The data that support the findings of this study are available on request from the corresponding author. The data are not publicly available due to privacy or ethical restrictions.
